# CK2-Mediated Hyperphosphorylation of Topoisomerase I Targets Serine 506, Enhances Topoisomerase I–DNA Binding, and Increases Cellular Camptothecin Sensitivity

**DOI:** 10.1371/journal.pone.0050427

**Published:** 2012-11-21

**Authors:** Keya Bandyopadhyay, Pingchuan Li, Ruth A. Gjerset

**Affiliations:** Torrey Pines Institute for Molecular Studies, San Diego, California, United States of America; Bauer Research Foundation, United States of America

## Abstract

Topoisomerase I is the target for a potent class of chemotherapeutic drugs derived from the plant alkaloid camptothecin that includes irinotecan and topotecan. In this study we have identified a novel site of CK2-mediated topoisomerase I (topo I) phosphorylation at serine 506 (PS506) that is relevant to topo I function and to cellular responses to these topo I-targeted drugs. CK2 treatment induced hyperphosphorylation of recombinant topo I and expression of the PS506 epitope, and resulted in increased binding of topo I to supercoiled plasmid DNA. Hyperphosphorylated topo I was approximately three times more effective than the basal phosphorylated enzyme at relaxing plasmid supercoils but had similar DNA cleavage activity once bound to DNA. The PS506 epitope was expressed in cancer cell lines with elevated CK2 activity, hyperphosphorylated topo I, and increased sensitivity to camptothecin. In contrast, PS506 was not detected in normal cells or cancer cell lines with lower levels of CK2 activity. By experimentally manipulating CK2 activity in cancer cell lines, we demonstrate a cause and effect relationship between CK2 activity, PS506 expression, camptothecin-induced cellular DNA damage, and cellular camptothecin sensitivity. Our results show that the PS506 epitope is an indicator of dysregulated, hyperphosphorylated topo I in cancer cells, and may thus serve as a diagnostic or prognostic biomarker and predict tumor responsiveness to widely used topo I-targeted therapies.

## Introduction

Topoisomerase I (topo I) plays an essential role in DNA synthesis by relaxing the torsional stress of DNA supercoils that form in front of the advancing replication fork [1], [2]. During the reaction, topo I binds to double-stranded DNA and catalyzes a single-strand cleavage, becoming covalently linked to one end of the break to form an intermediate structure termed the “cleavage complex.” Following DNA unwinding, topo I catalyzes break resealing and dissociates from the DNA (reviewed in reference [3]). The cleavage complex generated by topo I is the cellular target for a widely used and potent class of camptothecin-based chemotherapeutic drugs that includes irinotecan and topotecan. Binding of these drugs to the cleavage complex prevents resealing of the single-strand break, which becomes a lethal double-strand break upon encounter with the advancing replication fork [1], [4], [5]. Topo I activity is therefore essential for the camptothecin-based drugs to cause lethal DNA damage, and accordingly, camptothecin often has a greater effect on cells with higher topo I activity [6]–[10].

Topo I activity is influenced by phosphorylation, which primarily affects serine residues in vivo [11]–[13]. Several serine kinases have been implicated in topo I phosphorylation, including protein kinase C (PKC), cyclin-dependent kinase I (cdk-1), and protein kinase CK2 (formerly casein kinase II) [14], although the roles played by these enzymes in regulating topo I activity are not fully defined. While it is known that a basal level of phosphorylation is required for topo I activity [15], we found that a large fraction of cancer cell lines contain a more highly serine-phosphorylated population of topo I (hyperphosphorylated topo I) [6]. Moreover, the abundance of hyperphosphorylated topo I in these cells correlates with increased topo I DNA relaxation activity and cellular sensitivity to camptothecin compared to normal cell lines or cancer cell lines with lower levels of topo I serine phosphorylation [6]. Furthermore, we found that cancer cell lines with hyperphosphorylated topo I consistently express elevated levels of CK2, while levels of PKC and cdk-1 are variable across cell lines and do not consistently correlate with the hyperphosphorylation status of topo I [6]. Modulation of CK2 levels revealed a direct cause and effect relationship between elevated CK2, topo I hyperphosphorylation and increased activity, and increased cellular sensitivity to camptothecin [6]. These results indicated that CK2, an enzyme that is increasingly recognized as an important player in cancer [16], is a major regulator of topo I in human cancer cells, and the findings are consistent with other studies linking CK2 to topo I serine phosphorylation and camptothecin sensitivity in murine lymphoma cells [17], [18]. CK2-mediated regulation of topo I could therefore have broad relevance to the mechanism and treatment of cancer.

To better understand the significance of topo I hyperphosphorylation, we analyzed the residues targeted by CK2. Here, we provide evidence for a novel site of phosphorylation on topo I, serine 506 (PS506), which is present in cancer cells with elevated CK2, hyperphosphorylated topo I, and increased camptothecin sensitivity. The PS506 form of topo I is also generated in vitro by treatment of recombinant topo I with CK2 and exhibits increased DNA binding and DNA relaxation activity. Normal cell lines and cancer cell lines with lower levels of CK2 express a basal phosphorylated enzyme that lacks PS506; notably, these cell lines are more resistant to camptothecin. The PS506 epitope may therefore be associated with certain pathways of malignant transformation and may provide a biomarker for early detection or prognosis in cancer, as well as an indicator of tumor responsiveness to camptothecin-based therapeutics.

## Materials and Methods

### Cell Culture

The following cell lines were purchased from the American Type Culture Collection: H358 non-small cell lung cancer cells; PC-3, DU145, and LNCaP prostate cancer cells; HT29 and SW480 colon cancer cells; MDA-MB-436 and MDA-MB-231 breast cancer cells; and SKOV-3 and OVCAR-3 ovarian cancer cells. The OC3 esophageal adenocarcinoma cell line, derived from a metastatic lymph node of a Barrett’s esophageal lesion [19], was provided by Dr. Rebecca Fitzgerald (MRC Cancer Cell Unit, Hutchison-MRC Research Center, Cambridge, U.K.). The Hs27a human stromal cell line was derived from normal bone marrow and immortalized with the human papilloma virus type 16 E6/E7 genes [20] and was provided by Dr. Beverly Torok-Storb (Fred Hutchinson Cancer Research Center, Seattle). All cell lines except H358 were maintained in Dulbecco’s Modified Eagle’s Medium supplemented with 10% newborn calf serum, gentamycin, and non-essential amino acids. H358 cells were maintained in RPMI 1640 medium with the same supplements. Cell lines were tested to be free of mycoplasma using the Mycoplasma Detection Kit (InvivoGen, San Diego, CA).

### Hyperphosphorylation of Topo I by CK2 Treatment

With the exception of the His/FLAG-tagged human topo I expressed in H358 and SW480 cells (see below), all recombinant topo I used in this study was baculovirus-expressed human topo I (“R-topo I”) purchased from TopoGEN (Port Orange, FL). Hyperphosphorylated R-topo I for Western analyses and binding assays was generated by two consecutive treatments, each for 30 min at 37°C, with 10 units of CK2 (Promega, Madison, WI) per microgram of topo I, as described in the manufacturer’s instructions. For LC-MS determination of CK2 phosphorylation sites, R-topo I was first dephosphorylated by treatment for 30 min at 37°C with 10 units of agarose-immobilized alkaline phosphatase (Sigma-Aldrich, St. Louis, MO) per microgram of topo I, as described in the manufacturer’s instructions, followed by hyperphosphorylation with CK2.

### LC-MS Analysis of Hyperphosphorylated R-topo I

Samples of 1 µg R-topo I protein were dephosphorylated and hyperphosphorylated as described above, subjected to SDS-PAGE, and stained with colloidal Coomassie Blue (Invitrogen, Carlsbad, CA). The topo I band was excised and provided to the Mass Spectrometry Facility at the University of California, San Diego, where it was subjected to in-gel digestion with trypsin and processed as described in [21], before analysis using a QSTAR-Elite hybrid mass spectrometer. The data were analyzed by Dr. Majid Ghassemian, Director of the Mass Spectrometry Facility. Mass spectrometry tracings are supplied in Figure S1.

### Antibodies

The primary antibodies used were: goat polyclonal anti-topo I, mouse monoclonal anti-actin (both from Santa Cruz Biotechnology, Santa Cruz, CA), mouse monoclonal anti-phosphoserine (Sigma-Aldrich), rabbit polyclonal anti-histone H2A.X (Bethyl Laboratories, Montgomery, TX), and rabbit polyclonal anti–γ-H2A.X [ser139] (Novus Biologicals, Littleton, CO). The secondary antibodies used were goat anti-rabbit horseradish peroxidase (HRP), goat anti-mouse HRP, and donkey anti-goat HRP (Santa Cruz Biotechnology). For Westerns, primary and secondary antibodies were used at 1∶100 and 1∶1000 dilutions, respectively. Rabbit polyclonal antisera were generated to topo I peptides containing phosphorylated serine 506 (PS506) or non-phosphorylated serine 506 (S506) (sequence: TVGCCSLRVEHINLHPELKKC; serine 506 underlined) by Anaspec (Fremont, CA). IgG fractions were purified from the antisera and used in Westerns at 1∶100 dilution.

### Cell Viability Assay

Cells were added to 96-well plates at 2×10^3^ cells per well and incubated for 3 days, the first 18 h of which was in the presence of camptothecin (Sigma-Aldrich). Viability was measured 3 days after the start of camptothecin treatment by the MTS bioconversion assay, as previously described [6], [7]. In some experiments, cells were first exposed for 1 h to 10 µM of the specific CK2 inhibitor, tetrabromobenzotriazole (TBB; Calbiochem/EMD Millipore, Billerica, MA) and then to camptothecin for 18 h. In other experiments, cells were exposed concurrently to 10 nM of the specific CK2 activator 1-ethyl-4,5-dicarbamoyl imidazole and camptothecin for 18 h and then to the CK2 activator alone for the remaining incubation period. The CK2 activator was originally described in [22] and further described in [6]. The MTS assay was not affected by any of the reagents at the highest concentrations used.

### Topo I–plasmid DNA Relaxation Assay

Endogenous cellular topo I, transduced topo I gene products (described below), and R-topo I were assayed for their ability to relax supercoiled plasmid DNA using a Topo I Assay Kit (TopoGEN) as previously described [7]. Cellular topo I was released from nuclei of SKOV-3 and OVCAR-3 cells in high-salt buffer by a procedure adapted from reference [23]. Briefly, trypsinized and cold PBS-washed cells from one 10 cm culture dish (∼2.5 × 10^6^ cells) were suspended in 400 µl of cold hypotonic cell swelling buffer containing 10 mM Hepes pH 7.9, 10 mM KCl, 0.1 mM EDTA, 0.1 mM EGTA, 1 mM DTT, 0.5 mM PMSF, and complete protease inhibitors (Roche, Indianapolis, IN). Cells were incubated on ice for 15 min and then lysed by the addition of 0.6% NP-40 (EMD Millipore, Billerica, MA), followed by gentle pipetting. The lysate was centrifuged at 200 *g* for 5 min and the supernatant discarded. The nuclear pellet was extracted by addition of 100 µl of cold 20 mM Hepes (pH 7.9), 400 mM NaCl, 0.1 mM EDTA, 0.1 mM EGTA, 1 mM DTT, 0.5 mM PMSF, 10% (v/v) glycerol, followed by incubation on ice for 30 min with occasional gentle pipetting. The lysate was then centrifuged at 12,000 *g* for 10 min and the supernatant was used as nuclear extract. Relaxation assays were performed with 1–10 ng of R-topo I or with 0.75 µg of nuclear extract protein for assay of endogenous enzyme. For assays of SW480 transduced gene products selected on cobalt agarose (produced and selected as described below), an amount equivalent to 10 µg of starting nuclear extract protein was used.

### Protein Kinase CK2 Assay

CK2 activity was measured in whole cell lysates prepared as for Western analysis using an assay kit [(Upstate Biotechnology/Millipore (Temecula, CA)]. The assay was performed according to the manufacturer’s instructions and as described previously [6], using [γ-^33^P]-ATP (3000 Ci/mmol; MP Biomedicals, Solon, OH) and 10 µl (10 µg) cell lysate.

### Western Analysis

Whole cell lysates were prepared by addition of cold high-salt lysis buffer (20 mM Hepes pH 7.9, 400 mM NaCl, 1 mM EDTA, 1 mM EGTA, 1 mM DTT, 1 mM PMSF, 10% glycerol, and complete protease inhibitors) directly to the plates, followed by processing of lysates as previously described [6], [14]. Samples of 75 µg cellular protein or 0.5 µg R-topo I were resolved by SDS-PAGE and transferred to PVDF membranes. Membranes were immunostained with the appropriate antibodies used at the dilutions described above and visualized with ECL reagent (Amersham/GE Healthcare, Buckinghamshire, U.K.). In some experiments, band intensities were quantified digitally using an Alpha imager.

### Coimmunoprecipitation/Western Analyses

For detection of serine-phosphorylated endogenous topo I, whole cell lysates from 10^6^ cells were prepared as described for Western analysis and adjusted to a final concentration of 150 mM NaCl. Lysates were mixed with 10 µg of goat anti-topo I antibody (specific for topo I C-terminus) and immunoprecipitations were performed as previously described [24]. Samples were resolved by SDS-PAGE and Western blots were probed with anti-total topo I (control) or with purified IgG fractions from anti-PS506 and anti-S506 antisera, as indicated in the figures.

### Expression of Wild-type Topo I and Serine 506 to Alanine Mutant Topo I (A506)

The sequence of full-length wild-type human topo I, PCR amplified from H358 cell cDNA, was subcloned together with an N-terminal FLAG sequence form pCMV-Tag-1 (Stratagene, San Diego, CA) into the pTriEx-2 Hygro expression vector (EMD Millipore Biosciences), which supplies a His-tag coding sequence for selection of the gene product on cobalt agarose. The cloned sequence was verified by sequencing (Retrogen, San Diego, CA) and confirmed to be the complete wild-type human topo I coding sequence (GenBank sequence NM_003286). Site-directed mutagenesis of serine 506 to alanine (referred to as A506) was performed by primer extension according to procedures described in the Mutagenesis Application Guide available on the Integrated DNA Technologies website (www.idtdna.com), with primers synthesized by Integrated DNA Technologies (San Diego, CA): forward, ACTGTGGGCTGCTGCGCACTTCGTGTGGAG reverse, CTCCACACGAAGTGCGCAGCAGCCCACAGT. The cloned mutated sequence was verified by sequencing.

H358 and SW480 cells were transfected with the wild-type and A506 topo I expression vectors using TurboFect in vitro Transfection Reagent (Thermo Scientific, Rockford, IL) with 15 µg of vector per 10 cm plate (1 or 2 plates per transfection, ∼2.5×10^6^ cells per plate). Whole cell lysates were prepared 48 h later as described above for Western analysis. Transduced gene products were selected with HisPur™ Cobalt Resin (Thermo Scientific) according to the manufacturer’s procedure, and then used for Western analyses or plasmid relaxation assays, as described above.

### Topo I–plasmid DNA Non-covalent Binding Assay

To prepare radiolabeled plasmid (pCMV-FLAG; Sigma-Aldrich), a 5 ml culture of transformed XL-1 bacteria was grown overnight in the presence of 1 µCi [^3^H]-thymidine (Perkin Elmer, Waltham, MA). The plasmid was then purified and used as a substrate for R-topo I binding. Binding assays were performed with 0.03 pmol plasmid DNA (600 ng, 8770 cpm) and 0.3 pmol topo I in 50 µl low-salt buffer (75 mM NaCl, 10 mM Tris, pH 7.5) for 30 min at 4°C. These conditions allow non-covalent association of topo I with the plasmid DNA but prevent catalytic nicking, as described [25]. The topo I–DNA complexes were captured by adding 250 µl immunoprecipitation buffer (10 mM sodium phosphate pH 7.0, 75 mM NaCl, 0.1% SDS, 1% sodium deoxycholate, 0.5% NP40, and complete protease inhibitors), 2 µl goat anti-topo I IgG, and 10 µl protein-AG agarose for 1 h on ice. The immunoprecipitated complexes were then recovered by centrifugation and eluted from the protein-AG agarose at pH 2. The amount of co-immunoprecipitated DNA in the sample was quantified by scintillation counting.

### Topo I DNA Nicking Assay

A radiolabeled suicide substrate was prepared using the sequence and methods in reference [26], and further details are provided in Figure S2. The substrate is a 94 bp double-stranded hairpin structure constructed by ligation of 3 oligonucleotides, followed by slow annealing. The substrate was labeled internally with [^33^P] and contains a 16-nucleotide topo I binding and cleavage sequence identified in the rDNA spacers of *Tetrahymena* and *Dictyostelium* [27]. Cleavage by topo I occurs 3 bases upstream of the engineered nick in which the 5′-hydroxyl group required for resealing is replaced by a phosphate group. Therefore, once topo I has cleaved the suicide substrate, it remains trapped in a covalent complex with the DNA. To measure nicking activity, the reaction was initiated with non-covalent complexes that were preformed by incubating 0.3 pmol of untreated or CK2-treated topo I with 0.3 pmol radiolabeled suicide substrate (7000 cpm) in low-salt buffer (75 mM NaCl, 10 mM Tris, pH 7.5) for 30 minutes on ice. Equivalent amounts of preformed complexes were then incubated at 8°C for varying times and subjected to K^+^SDS precipitation, as originally described in reference [28]. Briefly, the topo I reaction (50 µl) was stopped by addition of 100 µl 2% SDS/2 mM EDTA and the mixture was heated at 65°C for 10 min. Covalently linked protein–DNA complexes were precipitated by addition of 50 µl 250 mM KCl and incubation on ice for 10 min. Precipitates were recovered by centrifugation and DNA was quantified by scintillation counting.

## Results

### CK2 Phosphorylates Serine 506 in Recombinant Human Topo I

To identify CK2-targeted sites in topo I, we dephosphorylated recombinant human topo I (R-topo I) with alkaline phosphatase and rephosphorylated it extensively with protein kinase CK2 to mimic in vivo hyperphosphorylation. The CK2-treated, gel-purified protein was digested with trypsin and subjected to mass spectrometry analysis. Two phosphopeptides were identified, one of which contained phosphorylated serine 112, a site in the N-terminal domain previously identified as a protein kinase cdk-1–targeted site not involved in regulation of topo I activity [14]. The second peptide contained phosphorylated serine 506 (PS506) (Figure S1), which is located in the core domain of the protein that interacts with DNA and encompasses the active site [29]. Serine 506 has not previously been reported as a phosphorylation site in topo I. We therefore investigated the involvement of PS506 in topo I DNA binding and catalytic activity, and its correlation with the topo I hyperphosphorylation observed in cancer cell lines expressing elevated CK2 levels.

**Figure 1:**
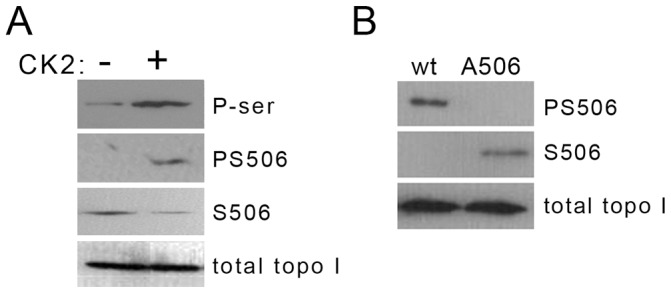
Western analysis of CK2-mediated phosphorylation of recombinant topo I. (**A**) Recombinant, baculovirus-expressed topo I was analyzed before or after treatment with CK2. Membranes were probed with antibodies to total phosphoserine (P-ser), PS506, S506, or total topo I. Each lane contains 0.5 µg protein. (**B**) His-tagged wild-type (wt) or ser506ala (A506) topo I gene products were analyzed with antibodies to PS506, S506, or total topo I. Gene products were selected on cobalt agarose from lysates of H358 cells 2 days after transduction. Each lane represents 2×10^6^ cell equivalents.

We raised rabbit polyclonal antisera to either a phosphoserine 506-containing topo I peptide (antiserum pAb506-P) or a non-phosphorylated topo I peptide encompassing S506 (pAb506). IgG fractions were purified from the antisera and their specificity was verified by Western blotting with R-topo I. We confirmed that R-topo I is basally serine-phosphorylated in the absence of CK2 treatment, as detected with a pan phosphoserine-specific antibody (Figure 1A, P-Ser). The basal phosphorylated protein lacks immunoreactivity with pAb506-P IgG (Figure 1A, PS506) but reacts with non-phospho-specific pAb506 IgG (Figure 1A, S506), indicating that the basal phosphorylation sites are distinct from PS506. Treatment of R-topo I with CK2 in vitro increased the overall level of serine phosphorylation and induced immunoreactivity with pAb506-P (Figure 1A, P-Ser and PS506). As expected, we observed inverse patterns of immunoreactivity with pAb506 and pAb506-P. That is, topo I reactivity with the non-phospho-specific IgG decreased following CK2 treatment whereas immunoreactivity with the phospho-specific IgG increased (Figure 1A, compare PS506 and S506 rows). These results indicate that the PS506 epitope is not present in R-topo I but can be induced by treatment with CK2.

PS506 can also be generated in vivo and is specifically recognized by pAb506-P (Figure 1B). H358 lung cancer cells, shown previously to express elevated CK2 and to hyperphosphorylate topo I [6], were transduced with expression vectors encoding His/FLAG-tagged wild-type topo I (wt) or topo I with a serine 506 to alanine point mutation (A506). The recombinant gene products were selected on cobalt agarose and analyzed by Western blotting for immunoreactivity with pAb506-P and pAb506. As shown in Figure 1B (top row), the wild-type transduced gene product, but not the A506 mutant, was immunoreactive with pAb506-P. An inverse pattern of immunoreactivity was observed with pAb506 (Figure 1B, 2nd row).

### Serine 506 Phosphorylation of Recombinant Topo I Enhances Relaxation of Supercoiled DNA by Increasing Topo I–DNA Binding

**Figure 2:**
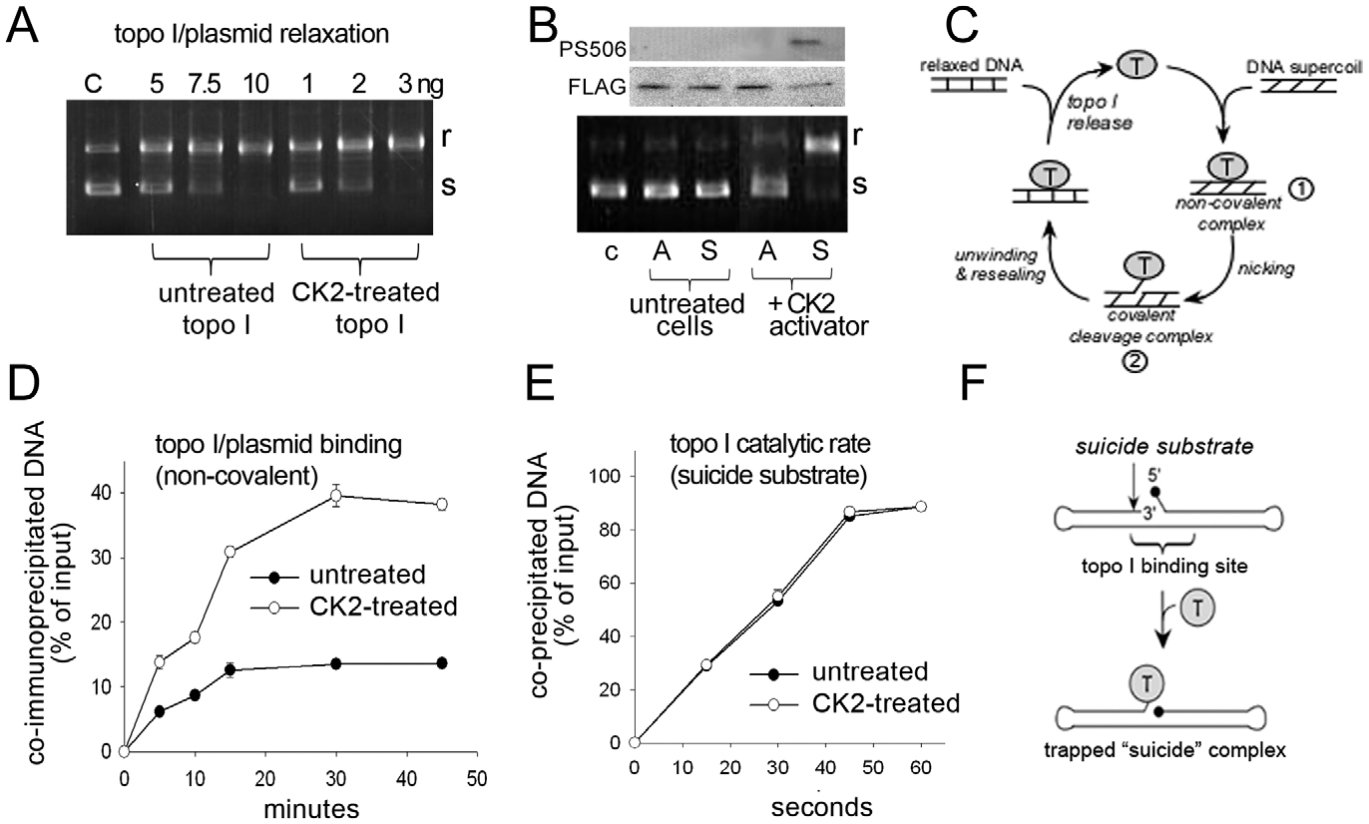
Effects of hyperphosphorylation on topo I binding, relaxation, and nicking activity. (A) Agarose gel electrophoresis of products of a supercoiled plasmid DNA relaxation assay carried out with basal or hyperphosphorylated R-topo I. Reactions contained 5, 7.5, or 10 ng of basal phosphorylated R-topo I, or 1, 2, or 3 ng of hyperphosphorylated R-topo I. C = untreated plasmid control, s = supercoiled DNA, r = relaxed DNA. (B) (Top) Western analysis of PS506 and FLAG expression in cobalt agarose-selected A506 (“A”) and wild-type (S506, “S”) gene products. Nuclear extracts of transduced SW480 cells were collected before or 2 days after treatment with the CK2 activator 1-ethyl-4,5-dicarbamoyl imidazole. Each lane represents 75 µg. (Bottom) Agarose gel showing results of a plasmid relaxation assay carried out with the same cobalt agarose-selected proteins. C = untreated control plasmid. (C) Schematic of the steps in topo I-mediated relaxation of DNA supercoils, involving non-covalent association of topo I with DNA (intermediate 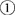) followed by catalytic single-strand nicking (intermediate 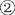). (D) Time course of non-covalent association of 0.3 pmol basal (•) or hyperphosphorylated (○) R-topo I to 0.03 pmol of radiolabeled plasmid DNA. Topo I–DNA complexes were recovered and DNA quantified by scintillation counting. Results show the % of input DNA present in DNA–topo I complexes. (E) Catalytic rate of basal (•) and hyperphosphorylated (○) R-topo I on a synthetic suicide substrate following the formation of non-covalent complexes at 4°C. (F) Diagram of hairpin structure of suicide substrate showing topo I cleavage sequence (↓) and blocked 5′-end carrying a phosphate group (•). Diagram adapted from reference [26] with permission from Oxford University Press.

To evaluate the functional consequences of serine 506 phosphorylation in vitro, we assayed the relaxation of supercoiled plasmid DNA by varying amounts of basal phosphorylated R-topo I or CK2-hyperphosphorylated R-topo I expressing PS506. The reaction products (supercoiled and relaxed forms of the plasmid) were separated by agarose gel electrophoresis based on their differential mobility (Figure 2A) and the bands were quantified digitally. We found that treatment of the supercoiled plasmid with 5, 7.5, or 10 ng of basal phosphorylated R-topo I (not CK2-treated) reduced supercoiled DNA levels by 44%, 62%, and 91%, respectively. Treatment of plasmid with 1, 2, or 3 ng of hyperphosphorylated R-topo I (CK2-treated) caused approximately the same decrease in supercoiled DNA levels (42%, 68%, and 99%, respectively). Thus, CK2-hyperphosphorylated R-topo I expressing PS506 displays a DNA relaxation activity about 3-fold greater than that of basal phosphorylated R-topo I.

To further evaluate the specific effect of serine 506 phosphorylation on topo I-mediated DNA relaxation, we compared the plasmid relaxation activities of the A506 and wild-type topo I (S506) gene products isolated from SW480 cells transduced with the His/FLAG expression vectors described in Figure 1B. SW480 colon cancer cells express basal phosphorylated endogenous topo I and low levels of CK2 [6]. Transduced cells were left untreated or treated with the CK2 activator 1-ethyl-4,5-dicarbamoyl imidazole, a compound previously shown to increase CK2 activity and promote hyperphosphorylation of topo I in treated cells [6]. Two days after transfection, the transduced gene products were selected on cobalt agarose, evaluated by Western analysis, and assayed for plasmid relaxation activity. Western blotting with the anti-PS506 antibody pAb506-P revealed that CK2 treatment induced PS506 expression in the wild-type (S506) but not the A506 gene product (Figure 2B, lanes S and A, respectively). Consistent with the lack of PS506 expression in basal phosphorylated topo I, anti-PS506 reactivity was not detected in the S506 gene product isolated from untreated cells (Figure 2B). The transduced gene products were also assayed for plasmid relaxation activity, which showed that the activity correlated with the PS506 status (Figure 2B, agarose gel). The data in Figure 2B show that (a) both the S506 and A506 gene products isolated from untreated cells have low levels of plasmid relaxation activity under our assay conditions, (b) the plasmid relaxation activity of the S506 gene product is enhanced by CK2 activator treatment of transduced cells and correlates with its increased PS506 expression, and (c) the plasmid relaxation activity of the A506 gene product is unaffected by treatment of transduced cells with the CK2 activator and correlates with the lack of PS506 expression.

We next determined whether the enhanced relaxation activity of hyperphosphorylated R-topo I resulted from an increased association with DNA (intermediate 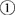, Figure 2C) or increased catalytic nicking activity once topo I was bound to DNA (intermediate 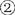, Figure 2C). To examine topo I DNA binding, we incubated radiolabeled plasmid DNA with basal phosphorylated or hyperphosphorylated R-topo I. The incubation was performed under low-salt conditions at 4°C to minimize complex dissociation and to prevent topo I nicking of DNA, as described [25]. Following incubation, the non-covalently bound topo I–DNA complexes (intermediate 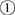) were captured by immunoprecipitation with an anti-topo I antibody, and the co-precipitated radiolabeled DNA was quantified by scintillation counting.

As shown in Figure 2D, the non-covalent association of basal phosphorylated R-topo I with plasmid DNA was maximal at 30 min incubation, at which point ∼13% of the input DNA was present in the topo I immunoprecipitate. In contrast, ∼40% of input DNA co-immunoprecipitated with hyperphosphorylated R-topo I (Figure 2C). We confirmed that the protein–DNA association was strictly non-covalent under these conditions in a second series of experiments in which the topo I–DNA complexes were precipitated using a K^+^SDS method [28] that dissociates non-covalent complexes and precipitates only DNA covalently linked to topo I. No DNA was recovered in the K^+^SDS precipitates (data not shown), indicating that the topo I–DNA complexes were non-covalently associated under our incubation conditions.

Having confirmed that hyperphosphorylation of topo I increased its binding to DNA, we next asked if hyperphosphorylation also enhanced the catalytic nicking rate of topo I (i.e., intermediate 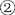, Figure 2C). We found that, in contrast to the topo I–DNA interaction, hyperphosphorylation of R-topo I did not affect its catalytic nicking rate. To show this, the catalytically cleaved intermediate 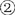 was captured using a 94-bp radiolabeled “suicide substrate” shown schematically in Figure 2F (structure design taken from ref [30], and further described in Materials and Methods and Figure S2). The suicide substrate contains a 16-nucleotide topo I binding and cleavage sequence (↓) but cannot religate because a 5′-phosphate group (•-) replaces the necessary 5′-hydroxyl. Hence, topo I remains covalently bound to the DNA substrate. Basal phosphorylated or hyperphosphorylated R-topo I was incubated with equimolar amounts of DNA (0.3 pmol each) for 30 minutes in low-salt conditions at 4°C. Under these conditions topo I remains catalytically inactive but all of the radiolabeled suicide substrate can be recovered in topo I immunoprecipitates (Figure S3). Preformed complexes were then incubated at 8°C to allow for catalysis and covalent linkage of topo I to the substrate. The covalently linked protein–DNA complexes were then precipitated with K^+^SDS, and radiolabeled DNA was quantified. As shown in Figure 2E, the recovery of radiolabeled suicide substrate in the K^+^SDS precipitate increased at the same rate for untreated and CK2-treated topo I. No reaction occurred during pre-incubation at 4°C, as indicated by the lack of measurable K^+^SDS-precipitated DNA at time 0 in Figure 2E. These results indicate that CK2-mediated hyperphosphorylation of serine 506 in R-topo I enhances its DNA relaxation activity by promoting increased association of topo I to DNA, but has no effect on topo I catalytic rate once bound to DNA.

### Elevated CK2 in Cancer Cells Increases Endogenous Topo I PS506 Expression and Relaxation Activity, and Cellular Camptothecin Sensitivity

**Figure 3:**
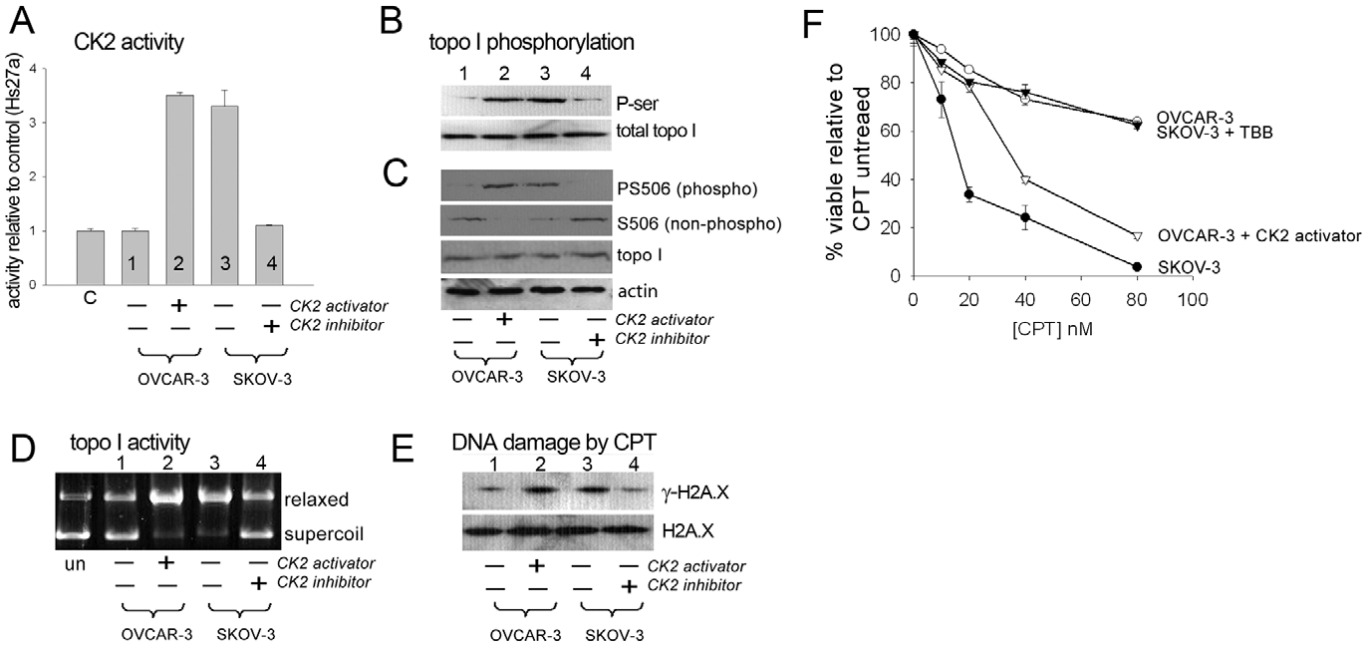
Modulation of CK2 activity and effects on topo I and cellular camptothecin responses. (**A**) CK2 activity measured in OVCAR-3 and SKOV-3 ovarian cancer cell lysates, before and after experimental modulation of CK2 levels. CK2 level in the control (C) Hs27a stromal cell line is shown for comparison. (**B**) Topo I serine phosphorylation status in OVCAR-3 and SKOV-3 cells before and after modulation of CK2 levels. Cell lysates (175 µg) were immunoprecipitated with goat anti-topo I C-terminal followed by Western analysis of phosphoserine (P-ser) or total topo I. (**C**) Western analysis of topo I PS506, S506, total topo I, and actin in lysates of OVCAR-3 and SKOV-3 cells before and after modulation of CK2 levels. Each lane represents 75 µg. (**D**) Topo I activity assayed by conversion of supercoiled plasmid DNA to the relaxed form by nuclear extracts prepared from OVCAR-3 or SKOV-3 cells before and after modulation of CK2 levels. First lane shows control, untreated plasmid. (**E**) Western analysis of γ-H2A.X expression as a marker of DNA damage in lysates of OVCAR-3 or SKOV-3 cells before or after modulation of CK2 levels, and following a 1 h exposure to 10 µM camptothecin (CPT). (**F**) Viability of OVCAR-3 or SKOV-3 cells on day 3 with or without experimental modulation of CK2, and following 18 h exposure to the indicated doses of camptothecin (CPT).

To determine if the expression of PS506 is related to endogenous topo I relaxation activity and cellular sensitivity to camptothecin in vivo, we examined two ovarian cancer cell lines with different endogenous CK2 levels. As shown in Figure 3A, CK2 activity in SKOV-3 cells was ∼3-fold higher than in OVCAR-3 cells or Hs27a mesenchymal cells (normal cell control, C). Treatment with the CK2 activator 1-ethyl-4,5-dicarbamoyl imidazole [6], [22] increased CK2 activity in OVCAR-3 cells to a level similar to that seen in untreated SKOV-3 cells. Conversely, CK2 activity in SKOV-3 cells was reduced to the levels seen in OVCAR-3 cells by treatment with the specific CK2 inhibitor 4,5,6,7-tetrabromobenzotriazole (TBB). Neither treatment affected cell growth over the 2–3 day incubations for these experiments (Figure S4).

To examine the phosphorylation state of endogenous topo I in these cells, topo I was immunoprecipitated from SKOV-3 and OVCAR-3 lysates and serine phosphorylation was examined by Western blotting. As shown in Figure 3B, we found that total serine phosphorylation of topo I in OVCAR-3 cells was low and correlated with the lower basal CK2 activity in these cells, but was enhanced by cell treatment with the CK2 activator (Figure 3B, lanes 1 and 2). Similarly, the high level of CK2 activity in SKOV-3 cells was reflected in the high level of topo I serine phosphorylation, which was reduced by inhibiting CK2 with TBB (Figure 3B, lanes 3 and 4). When the cell lysates were directly examined by Western blotting (Figure 3C), we found that expression of the PS506 epitope was low in OVCAR-3 cells but was enhanced by activation of CK2, and inversely, expression of the non-phosphorylated S506 epitope was readily detectable in untreated OVCAR-3 cells but was reduced following treatment with the CK2 activator (Figure 3C, lanes 1 and 2). Conversely, untreated SKOV-3 cells expressed high levels of the PS506 epitope that was suppressed by inhibiting CK2 (Figure 3C, lanes 3 and 4). In addition, the non-phosphorylated S506 epitope was barely detectable in untreated SKOV-3 cells but was enhanced by inhibiting CK2. Thus, virtually all of the topo I pool in SKOV-3 cells is phosphorylated on serine 506. These results show that the hyperphosphorylated form of topo I is generated by phosphorylation of serine 506, and confirm that CK2 is responsible for this phosphorylation.

To determine whether the PS506 status of cellular topo I correlates with total cellular topo I activity, we assayed DNA relaxation activity of topo I from nuclear lysates prepared before and after treatment of OVCAR-3 and SKOV-3 cells with modulators of CK2 activity. We found the OVCAR-3 nuclear lysate induced conversion of ∼15% supercoiled plasmid to the relaxed form, and this was increased to 90% following incubation of OVCAR-3 cells with the CK2 activator (Figure 3D, lanes 1 and 2). Conversely, the SKOV-3 nuclear lysate converted 90% of supercoiled plasmid to the relaxed form, and this was reduced to about 20% following TBB treatment of SKOV-3 cells (Figure 3D, lanes 3 and 4). Taken together with the mechanistic results presented in Figure 2, these results support a model in which the elevated levels of CK2 activity observed in cancer cells result in phosphorylation of topo I on serine 506, which in turn increases its DNA relaxation activity by enhancing the association of hyperphosphorylated topo I with DNA.

Because camptothecin-induced double-strand DNA breaks depend on topo I activity, the increased association of hyperphosphorylated, PS506-expressing topo I with DNA observed here would be expected to amplify DNA double-strand break formation in camptothecin-treated cells. To test this, we treated OVCAR-3 and SKOV-3 cells with the CK2 activator or inhibitor and examined the effect of camptothecin on expression of γ-H2A.X, a phosphorylated histone variant that increases in response to DNA double-strand break formation [31]. We found that camptothecin-mediated induction of γ-H2A.X in OVCAR-3 cells was increased following activation of CK2 (Figure 3E, lanes 1 and 2), and conversely, γ-H2A.X expression was high in SKOV-3 cells but was reduced following inhibition of CK2 (Figure 3E, lanes 3 and 4). Thus, CPT-induced expression of γ-H2A.X in both OVCAR-3 and SKOV-3 cells mirrored the respective cellular status of CK2 activity, topo I serine phosphorylation, PS506 expression, and topo I relaxation activity (Figure 3A–D).

These results suggested that direct manipulation of CK2 activity may therefore influence the cellular sensitivity to camptothecin through effects on topo I PS506 expression and activity. To examine this, we measured the viability of OVCAR-3 and SKOV-3 cells 3 days after treatment with varying doses of camptothecin. As shown in Figure 3F, the viability of SKOV-3 cells was virtually abolished by treatment with 80 nM camptothecin, while the same treatment had minimal effects on the viability of OVCAR-3 cells. The sensitivities of SKOV-3 and OVCAR-3 cells to camptothecin therefore correlated directly with the level of camptothecin-induced DNA damage and γ-H2A.X expression observed in Figure 3E. Treatment of SKOV-3 cells with TBB and OVCAR-3 cells with the CK2 activator rendered the cells less and more sensitive to camptothecin, respectively, also consistent with the induction of DNA damage observed in Figure 3E. These results thus revealed a functional relationship in vivo between high cellular CK2 levels, topo I hyperphosphorylation and the appearance of PS506, increased topo I relaxation activity, and elevated DNA damage in the presence of the topo I-targeted drug, camptothecin, all of which culminate in increased cellular sensitivity to camptothecin treatment.

### PS506 is Frequently Expressed in Cancer Cell Lines

**Figure 4:**
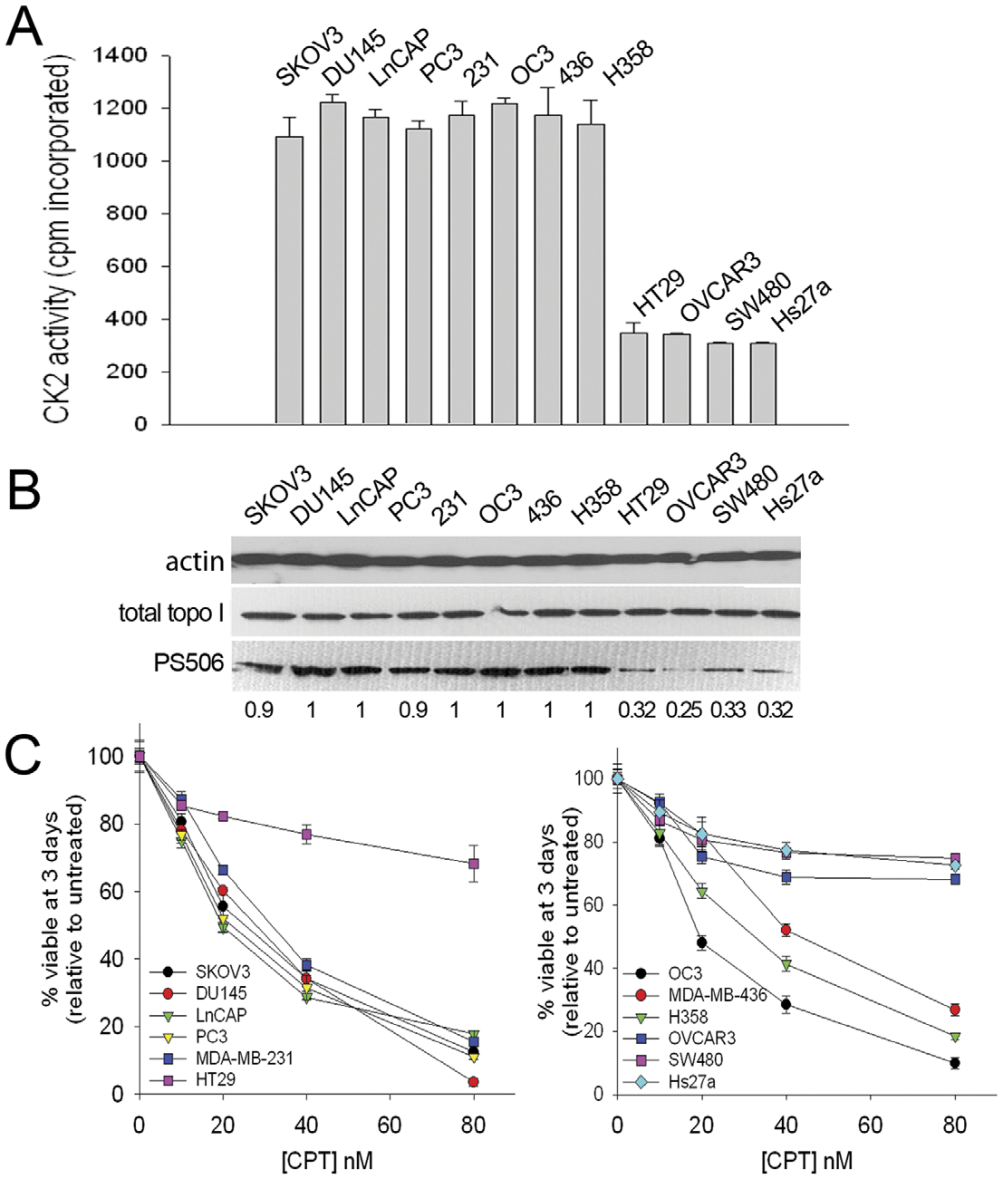
Correlation of CK2 activity, PS506 expression, and camptothecin sensitivity in cancer cell lines. (**A**) CK2 activity in cell lysates of the indicated cell lines. (**B**) Western analysis of PS506, total topo I, and actin in lysates of the indicated cell lines. Each lane contains 75 µg of cell lysate. Numbers below lanes refer to the intensities of the PS506 band relative to H358, as determined digitally. (**C**) Day 3 viability assays of the indicated cell lines following exposure to the indicated doses of camptothecin (CPT) for the first 18 h of the incubation.

To determine the prevalence of PS506 expression in cancer cell lines, we examined a panel of 11 cell lines of diverse origin for CK2 activity, PS506 expression, and camptothecin sensitivity. The mesenchymal stromal cell line Hs27a was used as a normal control. As shown in Figure 4A, CK2 activity in 8 of the 11 cancer cell lines was ∼3-fold higher than the activity in Hs27a cells. The same 8 cell lines also expressed PS506 at about 3–4-fold higher levels than Hs27a (Figure 4B). Three cell lines (HT29, OVCAR-3, and SW480) displayed lower levels of both CK2 activity and PS506 expression, which were similar to the levels in Hs27a cells (Figure 4A,B). All of the 8 cell lines with high CK2 activity and PS506 expression were also sensitive to camptothecin, with viabilities of 20% or less on day 3 following treatment with 80 nM camptothecin (Figure 4C). As expected, the 3 lines with reduced CK2 activity and PS506 expression were resistant to 80 nM camptothecin (Figure 4C). These results indicate that PS506 expression is common in cancer cell lines and correlates with increased camptothecin sensitivity.

## Discussion

In this study, we have identified a novel site of CK2-mediated topo I phosphorylation at serine 506 (PS506) that is relevant not only to topo I function but also to cellular responses to topo I-targeted drugs. CK2 treatment of R-topo I induced expression of the PS506 epitope and increased binding of the hyperphosphorylated topo I to supercoiled plasmid DNA. The hyperphosphorylated topo I was about 3 times more effective than the basal phosphorylated enzyme at relaxing plasmid supercoils, but had similar DNA cleavage activity once bound to DNA. The PS506 epitope was expressed in cancer cell lines with elevated CK2 activity, hyperphosphorylated topo I, and increased sensitivity to camptothecin compared to normal cells or cancer cells with normal levels of CK2. By experimentally manipulating CK2 activity in cancer cell lines, we demonstrated a cause and effect relationship between CK2 activity, PS506 expression, camptothecin-induced cellular DNA damage, and cellular camptothecin sensitivity. These results extend our previous observations that correlated CK2-mediated hyperphosphorylation of topo I with increased sensitivity to camptothecin [6] and are consistent with other reports linking CK2 and topo I activity [17], [18].

Our results demonstrate that the PS506 epitope is an indicator of dysregulated, hyperphosphorylated topo I in cancer cells, and may thus serve as a diagnostic or prognostic biomarker and predict tumor responsiveness to widely used topo I-targeted therapies such as irinotecan and topotecan. These drugs were FDA approved in 1996 for the treatment of advanced colon and ovarian cancers, respectively, and have subsequently demonstrated efficacy in a variety of other cancers, including breast, esophagus, pancreas, lung, head and neck, bone, cervix, and brain cancer [32], [33]. Furthermore, because topotecan crosses the blood-brain barrier, it has shown efficacy against brain metastases [34] and pediatric brain neoplasms [35]. Although these drugs are highly effective in some patients, tumor responses are variable and unpredictable, and at present there are no reliable tools to predict responses in individual patients. Further work will be needed to establish the prevalence and distribution of PS506 in tumor specimens, as well as the relative contribution of topo I dysregulation to clinical therapy responses, compared with other clinical factors. However, the PS506 biomarker could provide an additional tool to assess probable tumor responsiveness to topo I-targeted drugs prior to treatment, thereby facilitating treatment decision-making and improving treatment outcomes.

Overexpression of CK2 activity has been observed in many human cancers, including those of the colon, breast, prostate, lung, head and neck, and kidney [36]–[41], and may contribute to the underlying mechanism of cancer. CK2 has been shown to collaborate with oncogenes to promote cellular transformation in model systems, and the increased CK2 activity observed in human cancer correlates with increased dysplasia, tumor aggressiveness, and poor prognosis [37], [39], [42]–[45], suggesting a direct involvement of CK2 in pathways that underlie tumor progression. Although the precise mechanism(s) by which CK2 overexpression contributes to cancer is not well understood, the present study raises the possibility that dysregulation of topo I may be an important consequence of CK2 overexpression. Topo I, as an enzyme capable of DNA nicking and religation, has recombinogenic potential [46]–[48] and has been implicated in the mechanisms affecting genome stability [49], [50]. It is possible that the increased DNA binding capacity of the PS506-hyperphosphorylated form of topo I may cause the enzyme to associate aberrantly with chromosomal sites it does not normally occupy and subsequently to induce aberrant DNA nicking. If so, detection of the PS506 epitope could identify cells with increased potential for chromosomal rearrangements, translocations, or deletions that could drive malignancy.

Topo I is a 765-amino acid protein containing 4 domains defined through a combination of approaches, including sequence alignment, analysis of proteolytic fragments, and crystallography (see [3] for review). The domains include a poorly conserved N-terminal domain (residues 1–215) that is not required for enzyme activity, a highly conserved DNA-binding core domain containing most of the catalytic residues (residues 215 to 636), a poorly conserved linker domain (residues 636 to 713), and a conserved C-terminal domain (residues 713 to 765) that contains the active site tyrosine 723. Serine 506 is located in the DNA-binding core domain of topo I, between DNA-binding residues at positions 489, 490, 491, 501, and 533 identified by crystallography [29]. Although serine 506 does not form contacts DNA, its phosphorylation could potentially alter the conformation of this region and affect DNA binding by other residues in the domain. Phosphorylation of serine 506 could also indirectly affect topo I binding to DNA by promoting its association with other DNA-binding proteins. For example, topo I is known to interact with the p14 Alternate Reading Frame tumor suppressor protein (ARF) [7], [51], [52], a positively charged DNA binding protein that is also elevated in many cancers. Moreover, phosphorylation of topo I enhances its binding to ARF and increases its plasmid relaxation activity [7].

In addition to serine 506, we found serine 112 was phosphorylated in CK2-treated R-topo I. This site has previously been identified as a cdk-1 target in topo I from A549 human lung cancer cells and K562 human leukemia cells [14], although in that study the phosphorylation status of serine 112 was not found to affect topo I activity. We have shown that A549 and K562 cells do not overexpress CK2 [6], suggesting that serine 112 phosphorylation may be a feature of the basal phosphorylated enzyme and may not contribute to the properties of hyperphosphorylated topo I seen in the present study. However, further studies will be needed to clarify the significance of CK2-mediated serine 112 phosphorylation of topo I, including whether CK2 affects the overall pool size of topo I molecules phosphorylated on this site.

This is the first report of a CK2-targeted phosphorylation site at serine 506 of topo I. The finding that the PS506 epitope is unique to hyperphosphorylated topo I, together with our earlier observations that hyperphosphorylated topo I was not observed in cell lines derived from normal tissue [6], suggests that the PS506 epitope may be an abnormal phosphorylation characteristic of cancer cells with elevated CK2. This site could therefore have clinical relevance as a diagnostic marker and may shed light on the role of topo I dysregulation in malignancy. Further studies are ongoing to clarify the contribution of PS506 to malignant transformation and to determine how its expression varies with tumor type, tumor stage, and tumor response to therapy.

## Supporting Information

Figure S1 **Mass Spectrometry tracing. (A)** Table of predicted product ion masses (+1 charge) for the partially tryptic peptide fragment EEGETADTVGCCS[P]L with both cysteines carbamidomethylated. **(B)** Product ion scan spectrum for m/z = 1605.66. The predicted y and b ion series are mapped to the masses on the spectrum. The matched masses are highlighted in green in (A).
(DOCX)

Figure S2 **Description of suicide substrate used in** **Figure 2E** **. (A)** The sequence of the synthetic suicide substrate (Figure adapted from reference (a) with permission from Oxford University Press). The substrate was a design taken from reference (a) that traps topoisomerase in a covalent complex with DNA. The substrate is a 193-base synthetic DNA that contains a 16-nucleotide topo I binding and cleavage sequence identified in the rDNA spacers of *Tetrahymena* and *Dictyostelium* (reference b). Religation of DNA and release of topo I cannot occur due to the lack of a 5′-hydroxyl group on the DNA. The substrate is formed by ligating 3 oligonucleotides (denoted OL1, OL2, OL3), then slowly annealing the ligated products to form a hairpin structure. OL1 provides the 3′ end of the ligated product and OL3 provides the 5′ end. OL1 was 5′-phosphorylated with [γ-^33^P]-ATP and T4 kinase prior to annealing and ligation to allow tracking of covalent protein–DNA complexes (indicated by *) and OL2 was 5′-phosphorylated with unlabeled ATP and T4 kinase prior to ligation. After 5′-phosphorylation of OL1 and OL2, the 3 oligos were precipitated, resuspended at 50 pmol/µl in 10 mM Tris (pH 8) and 1 mM EDTA, and 1 µl of each oligo was annealed in 100 µl of 10 mM sodium phosphate (pH 7) and 150 mM sodium chloride. The mixture was heated to 95°C to achieve complete denaturation, then slowly cooled to 25°C (2°C decrease per min). T4 polynucleotide ligase was added (1200 units; New England BioLabs), the mixture was incubated for an additional 3–4 days at 4°C, and the product was then treated with T4 kinase and ATP, as described in reference (a). **(B)** Schematic showing the final product, a double-stranded hairpin structure of 94 bp in length and phosphorylated at the 5′ end. The topo I cleavage site (▾) lies 3 nucleotides upstream of the engineered nick in which the 5′-hydroxyl group required for resealing is replaced by a phosphate group (•). **(C)** 10% TBE PAGE analysis validating the accuracy of annealing: we showed that BamH1 digestion produced fragments of 50 and 20 bp, as predicted from the location of BamH1 sites in the sequence (▿ in Figure S1B). (a) Soe, k., Dianov, G., Nasheuer, H. P., Bohr, V. A., Grosse, F., and Stevnsner, T. A human topoisomerase I cleavage complex is recognized by an additional human topoisomerase I molecule in vitro. Nucleic Acids Res, *29:* 3195–3203, 2001. (b) Stevnsner, T., Mortensen, U. H., Westergaard, O., and Bonven, B. J. Interactions between eukaryotic DNA topoisomerase I and a specific binding sequence. J biol Chem, *264:* 10110–10113, 1989.
(DOCX)

Figure S3 **Demonstration that non-covalent binding of topo I to suicide substrate is complete at 30 minutes.** A sample of 0.3 pmol of untreated or CK2-treated recombinant baculovirus-expressed topo I (see Materials and Methods for CK2 treatment conditions) was incubated for 30 min at 4°C with 0.3 pmol (6200 cpm) of [^33^P]-radiolabeled suicide substrate (described in Figure S1) in 10 mM Tris (pH 7.5) and 75 mM NaCl. The protein–DNA complex was then immunoprecipitated with goat anti-topo I antibody, as described in Materials and Methods. The fraction of input radiolabeled DNA recovered in the immunoprecipitate was determined by scintillation counting and showed that binding was complete for both topo I species by 30 minutes.
(DOCX)

Figure S4 **Demonstration that growth rates of SKOV3 and OVCAR3 cells are unaffected by TBB or CK2 activator treatments.** Cells were plated in duplicate at 2×10^3^/well in 96-well plates. One day later, SKOV3 cells were treated for 1 h with 10 µM TBB or were left untreated. OVCAR3 cells were treated with 10 nM CK2 activator for the duration of the assay or were left untreated. On days 2–5, cells were pulsed for 6 h with 0.5 µCi/well [3H]-thymidine, harvested onto filters with a Brandel Harvester, and subjected to scintillation counting.
(DOCX)

## References

[1] Pommier Y, Pourquier P, Fan Y, Strumberg D (1998) Mechanism of action of eukaryotic DNA topoisomerase I and drugs targeted to the enzyme. Biochim Biophys Acta 1400: 83–105. 9748515 10.1016/s0167-4781(98)00129-8

[2] Wang JC (1996) DNA topoisomerases. Annu Rev Biochem 65: 635–692. 8811192 10.1146/annurev.bi.65.070196.003223

[3] Champoux JJ (2001) DNA topoisomerases: structure, function, and mechanism. Annu Rev Biochem 70: 369–413. 11395412 10.1146/annurev.biochem.70.1.369

[4] Tsao YP, Russo A, Nyamuswa G, Silber R, Liu LF (1993) Interaction between replication forks and topoisomerase I-DNA cleavable complexes: studies in a cell-free SV40 DNA replication system. Cancer Res 53: 5908–5914. 8261402

[5] Liu LF (1989) DNA topoisomerase poisons as antitumor drugs. Annu Rev Biochem 58: 351–375. 2549853 10.1146/annurev.bi.58.070189.002031

[6] Bandyopadhyay K, Gjerset RA (2011) Protein kinase CK2 is a central regulator of topoisomerase I hyperphosphorylation and camptothecin sensitivity in cancer cell lines. Biochemistry 50: 704–714. 21182307 10.1021/bi101110e PMC3046806

[7] Bandyopadhyay K, Lee C, Haghighi A, Baneres JL, Parello J, et al (2007) Serine phosphorylation-dependent coregulation of topoisomerase I by the p14ARF tumor suppressor. Biochemistry 46: 14325–14334. 18004878 10.1021/bi7013618

[8] Sugimoto Y, Tsukahara S, Oh-hara T, Isoe T, Tsuruo T (1990) Decreased expression of DNA topoisomerase I in camptothecin-resistant tumor cell lines as determined by a monoclonal antibody. Cancer Res 50: 6925–6930. 2170010

[9] Dingemans AM, Pinedo HM, Giaccone G (1998) Clinical resistance to topoisomerase-targeted drugs. Biochim Biophys Acta 1400: 275–288. 9748627 10.1016/s0167-4781(98)00141-9

[10] Larsen AK, Skladanowski A (1998) Cellular resistance to topoisomerase-targeted drugs: from drug uptake to cell death. Biochim Biophys Acta 1400: 257–274. 9748618 10.1016/s0167-4781(98)00140-7

[11] Coderoni S, Paparelli M, Gianfranceschi GL (1990) Phosphorylation sites for type N II protein kinase in DNA-topoisomerase I from calf thymus. Int J Biochem 22: 737–746. 2169438 10.1016/0020-711x(90)90009-r

[12] Pommier Y, Kerrigan D, Hartman KD, Glazer RI (1990) Phosphorylation of mammalian DNA topoisomerase I and activation by protein kinase C. J Biol Chem. 265: 9418–9422. 2160979

[13] Turman MA, Douvas A (1993) A casein kinase type II (CKII)-like nuclear protein kinase associates with, phosphorylates, and activates topoisomerase I. Biochem Med Metab Biol. 50: 210–225. 10.1006/bmmb.1993.1063 8260198

[14] Hackbarth JS, Galvez-Peralta M, Dai NT, Loegering DA, Peterson KL, et al (2008) Mitotic phosphorylation stimulates DNA relaxation activity of human topoisomerase I. J Biol Chem. 283: 16711–16722. 10.1074/jbc.M802246200 PMC2423254 18408216

[15] Coderoni S, Paparelli M, Gianfranceschi GL (1990) Role of calf thymus DNA-topoisomerase I phosphorylation on relaxation activity expression and on DNA-protein interaction. Role of DNA-topoisomerase I phosphorylation. Mol Biol Rep 14: 35–39. 2161075 10.1007/BF00422713

[16] Trembley JH, Wang G, Unger G, Slaton J, Ahmed K (2009) Protein kinase CK2 in health and disease: CK2: a key player in cancer biology. Cell Mol Life Sci 66: 1858–1867. 19387548 10.1007/s00018-009-9154-y PMC4385580

[17] Staron K, Kowalska-Loth B, Nieznanski K, Szumiel I (1996) Phosphorylation of topoisomerase I in L5178Y-S cells is associated with poly(ADP-ribose) metabolism. Carcinogenesis 17: 383–387. 8631120 10.1093/carcin/17.3.383

[18] Staron K, Kowalska-Loth B, Zabek J, Czerwinski RM, Nieznanski K, et al (1995) Topoisomerase I is differently phosphorylated in two sublines of L5178Y mouse lymphoma cells. Biochim Biophys Acta 1260: 35–42. 7999792 10.1016/0167-4781(94)00175-3

[19] Barry OP, Mullan B, Sheehan D, Kazanietz MG, Shanahan F, et al (2001) Constitutive ERK1/2 activation in esophagogastric rib bone marrow micrometastatic cells is MEK-independent. J Biol Chem 276: 15537–15546. 11297525 10.1074/jbc.M010847200

[20] Roecklein BA, Torok-Storb B (1995) Functionally distinct human marrow stromal cell lines immortalized by transduction with the human papilloma virus E6/E7 genes. Blood 85: 997–1005. 7849321

[21] Shevchenko A, Wilm M, Vorm O, Mann M (1996) Mass spectrometric sequencing of proteins silver-stained polyacrylamide gels. Anal Chem 68: 850–858. 8779443 10.1021/ac950914h

[22] Reikhardt BA, Kulikova OG, Borisova GY, Aleksandrova IY, Sapronov NS (2003) Status of the “protein kinase CK2-HMG14” system in age-related amnesia in rats. Neurosci Behav Physiol 33: 799–804. 14635996 10.1023/a:1025101516128

[23] Olnes MI, Kurl RN (1994) Isolation of nuclear extracts from fragile cells: a simplified procedure applied to thymocytes. Biotechniques 17: 828–829. 7840956

[24] Lee C, Smith BA, Bandyopadhyay K, Gjerset RA (2005) DNA damage disrupts the p14ARF-B23(nucleophosmin) interaction and triggers a transient subnuclear redistribution of p14ARF. Cancer Res 65: 9834–9842. 16267006 10.1158/0008-5472.CAN-05-1759

[25] McConaughy BL, Young LS, Champoux JJ (1981) The effect of salt on the binding of the eucaryotic DNA nicking-closing enzyme to DNA and chromatin. Biochim Biophys Acta 655: 1–8. 6266479 10.1016/0005-2787(81)90059-9

[26] Soe K, Dianov G, Nasheuer HP, Bohr VA, Grosse F, et al (2001) A human topoisomerase I cleavage complex is recognized by an additional human topisomerase I molecule in vitro. Nucleic Acids Res 29: 3195–3203. 11470877 10.1093/nar/29.15.3195 PMC55829

[27] Stevnsner T, Mortensen UH, Westergaard O, Bonven BJ (1989) Interactions between eukaryotic DNA topoisomerase I and a specific binding sequence. J Biol Chem 264: 10110–10113. 2542321

[28] Liu LF, Rowe TC, Yang L, Tewey KM, Chen GL (1983) Cleavage of DNA by mammalian DNA topoisomerase II. J Biol Chem 258: 15365–15370. 6317692

[29] Redinbo MR, Stewart L, Kuhn P, Champoux JJ, Hol WG (1998) Crystal structures of human topoisomerase I in covalent and noncovalent complexes with DNA. Science 279: 1504–1513. 9488644 10.1126/science.279.5356.1504

[30] Soe K, Grosse F (2003) p53 stimulates human topoisomerase I activity by modulating its DNA binding. Nucleic Acids Res 31: 6585–6592. 14602918 10.1093/nar/gkg846 PMC275542

[31] Rogakou EP, Boon C, Redon C, Bonner WM (1999) Megabase chromatin domains involved in DNA double-strand breaks in vivo. J Cell Biol 146: 905–916. 10477747 10.1083/jcb.146.5.905 PMC2169482

[32] Rothenberg ML (2001) Irinotecan (CPT-11): recent developments and future directions–colorectal cancer and beyond. Oncologist 6: 66–80. 11161230 10.1634/theoncologist.6-1-66

[33] National Comprehensive Cancer Network website. Available: www.NCCN.org. Accessed 2012 Oct 25.

[34] Wong ET, Berkenblit A (2004) The role of topotecan in the treatment of brain metastases. Oncologist 9: 68–79. 14755016 10.1634/theoncologist.9-1-68

[35] Bomgaars L, Berg SL, Blaney SM (2001) The development of camptothecin analogs in childhood cancers. Oncologist 6: 506–516. 11743213 10.1634/theoncologist.6-6-506

[36] Daya-Makin M, Sanghera JS, Mogentale TL, Lipp M, Parchomchuk J, et al (1994) Activation of a tumor-associated protein kinase (p40TAK) and casein kinase 2 in human squamous cell carcinomas and adenocarcinomas of the lung. Cancer Res 54: 2262–2268. 7513612

[37] Faust RA, Gapany M, Tristani P, Davis A, Adams GL, et al (1996) Elevated protein kinase CK2 activity in chromatin of head and neck tumors: association with malignant transformation. Cancer Lett 101: 31–35. 8625279 10.1016/0304-3835(96)04110-9

[38] Landesman-Bollag E, Romieu-Mourez R, Song DH, Sonenshein GE, Cardiff RD, et al (2001) Protein kinase CK2 in mammary gland tumorigenesis. Oncogene 20: 3247–3257. 11423974 10.1038/sj.onc.1204411

[39] Lin KY, Tai C, Hsu JC, Li CF, Fang CL, et al (2011) Overexpression of nuclear protein kinase CK2 alpha catalytic subunit (CK2alpha) as a poor prognosticator in human colorectal cancer. PLoS One 6: e17193. 21359197 10.1371/journal.pone.0017193 PMC3040762

[40] Stalter G, Siemer S, Becht E, Ziegler M, Remberger K, et al (1994) Asymmetric expression of protein kinase CK2 subunits in human kidney tumors. Biochem Biophys Res Commun 202: 141–147. 8037705 10.1006/bbrc.1994.1904

[41] Yenice S, Davis AT, Goueli SA, Akdas A, Limas C, et al (1994) Nuclear casein kinase 2 (CK-2) activity in human normal, benign hyperplastic, and cancerous prostate. Prostate 24: 11–16. 7507238 10.1002/pros.2990240105

[42] Gapany M, Faust RA, Tawfic S, Davis A, Adams GL, et al (1995) Association of elevated protein kinase CK2 activity with aggressive behavior of squamous cell carcinoma of the head and neck. Mol Med 1: 659–666. 8529132 PMC2229971

[43] Laramas M, Pasquier D, Filhol O, Ringeisen F, Descotes JL, et al (2007) Nuclear localization of protein kinase CK2 catalytic subunit (CK2alpha) is associated with poor prognostic factors in human prostate cancer. Eur J Cancer 43: 928–934. 17267203 10.1016/j.ejca.2006.11.021

[44] O-Charoenrat P, Rusch V, Talbot SG, Sarkaria I, Viale A, et al (2004) Casein kinase II alpha subunit and C1-inhibitor are independent predictors of outcome in patients with squamous cell carcinoma of the lung. Clin Cancer Res 10: 5792–5803. 15355908 10.1158/1078-0432.CCR-03-0317

[45] Faust RA, Niehans G, Gapany M, Hoistad D, Knapp D, et al (1999) Subcellular immunolocalization of protein kinase CK2 in normal and carcinoma cells. Int J Biochem Cell Biol 31: 941–949. 10533285 10.1016/s1357-2725(99)00050-3

[46] Shuman S (1989) Vaccinia DNA topoisomerase I promotes illegitimate recombination in Escherichia coli. Proc Natl Acad Sci U S A 86: 3489–3493. 2542933 10.1073/pnas.86.10.3489 PMC287163

[47] Shuman S (1992) DNA strand transfer reactions catalyzed by vaccinia topoisomerase I. J Biol Chem. 267: 8620–8627. 1314832

[48] Zhu J, Schiestl RH (1996) Topoisomerase I involvement in illegitimate recombination in Saccharomyces cerevisiae. Mol Cell Biol 16: 1805–1812. 8657156 10.1128/mcb.16.4.1805 PMC231167

[49] Arlt MF, Glover TW (2010) Inhibition of topoisomerase I prevents chromosome breakage at common fragile sites. DNA Repair (Amst) 9: 678–689. 20413351 10.1016/j.dnarep.2010.03.005 PMC2896008

[50] Tuduri S, Crabbe L, Conti C, Tourriere H, Holtgreve-Grez H, et al (2009) Topoisomerase I suppresses genomic instability by preventing interference between replication and transcription. Nat Cell Biol 11: 1315–1324. 19838172 10.1038/ncb1984 PMC2912930

[51] Ayrault O, Karayan L, Riou JF, Larsen CJ, Seite P (2003) Delineation of the domains required for physical and functional interaction of p14ARF with human topoisomerase I. Oncogene. 22: 1945–1954. 10.1038/sj.onc.1206214 12673200

[52] Karayan L, Riou JF, Seite P, Migeon J, Cantereau A, et al (2001) Human ARF protein interacts with topoisomerase I and stimulates its activity. Oncogene 20: 836–848. 11314011 10.1038/sj.onc.1204170

