# Supplementary figures and images for: CK2-Mediated Hyperphosphorylation of Topoisomerase I Targets Serine 506, Enhances Topoisomerase I–DNA Binding, and Increases Cellular Camptothecin Sensitivity
Source: PLoS One. 2012 Nov 21;7(11):e50427. doi: 10.1371/journal.pone.0050427 (PMC3503890; doi:10.1371/journal.pone.0050427)

**Figure S1**

**
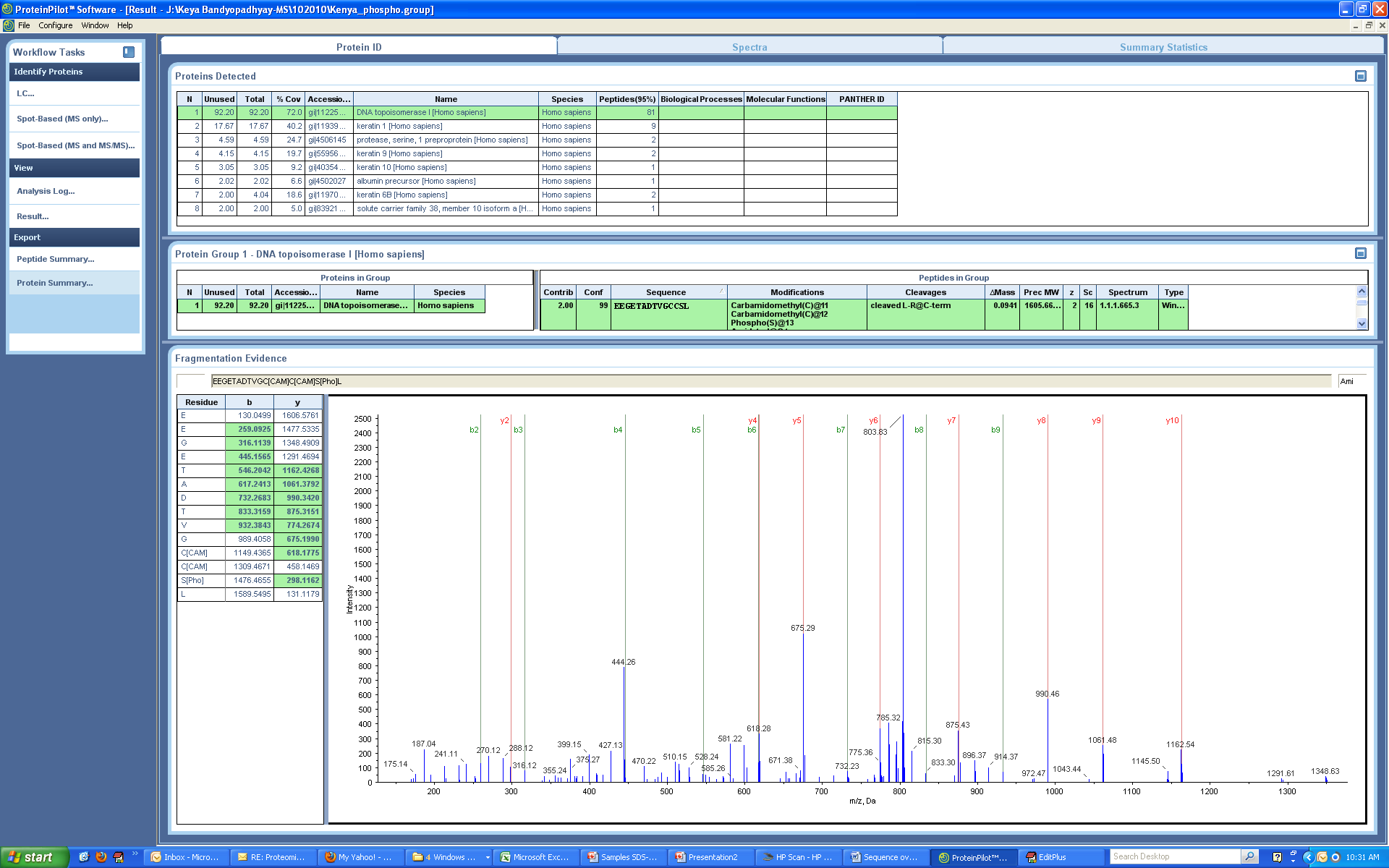
Mass spectrometry tracing**

**A**

**B**


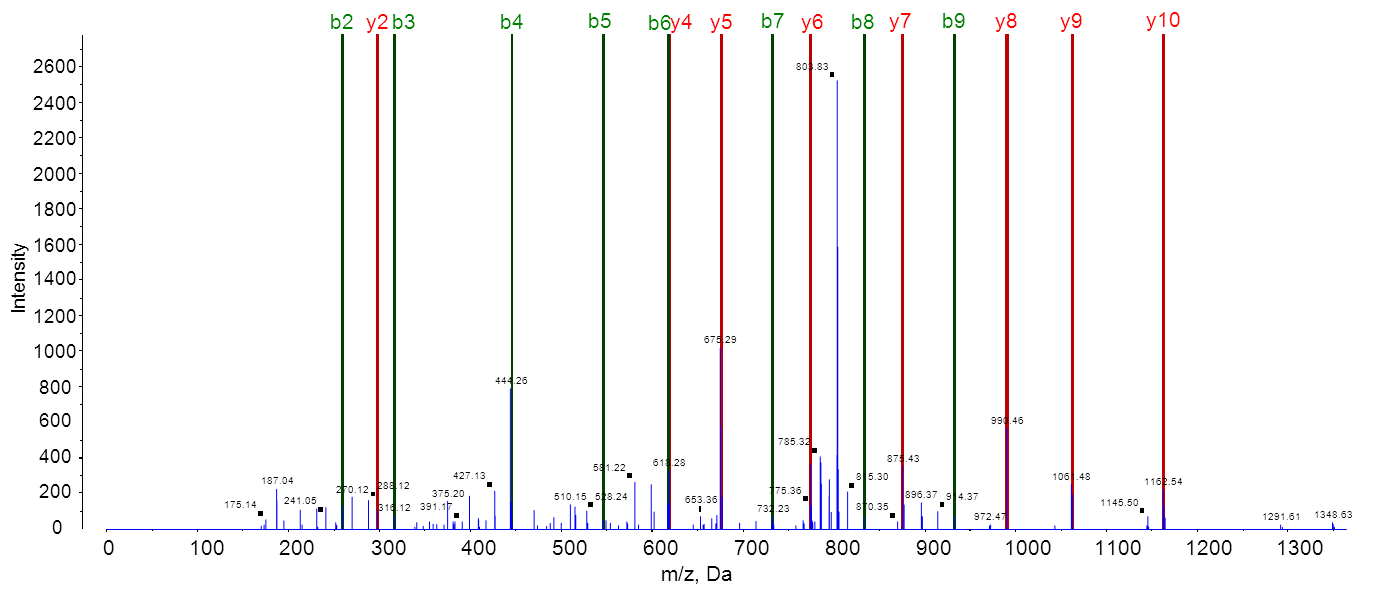

Supplement: Figure S1 — Mass Spectrometry tracing. (A) Table of predicted product ion masses (+1 charge) for the partially tryptic peptide fragment EEGETADTVGCCS[P]L with both cysteines carbamidomethylated. (B) Product ion scan spectrum for m/z = 1605.66. The predicted y and b ion series are mapped to the masses on the spectrum. The matched masses are highlighted in green in (A). (DOCX) [file pone.0050427.s001.docx]

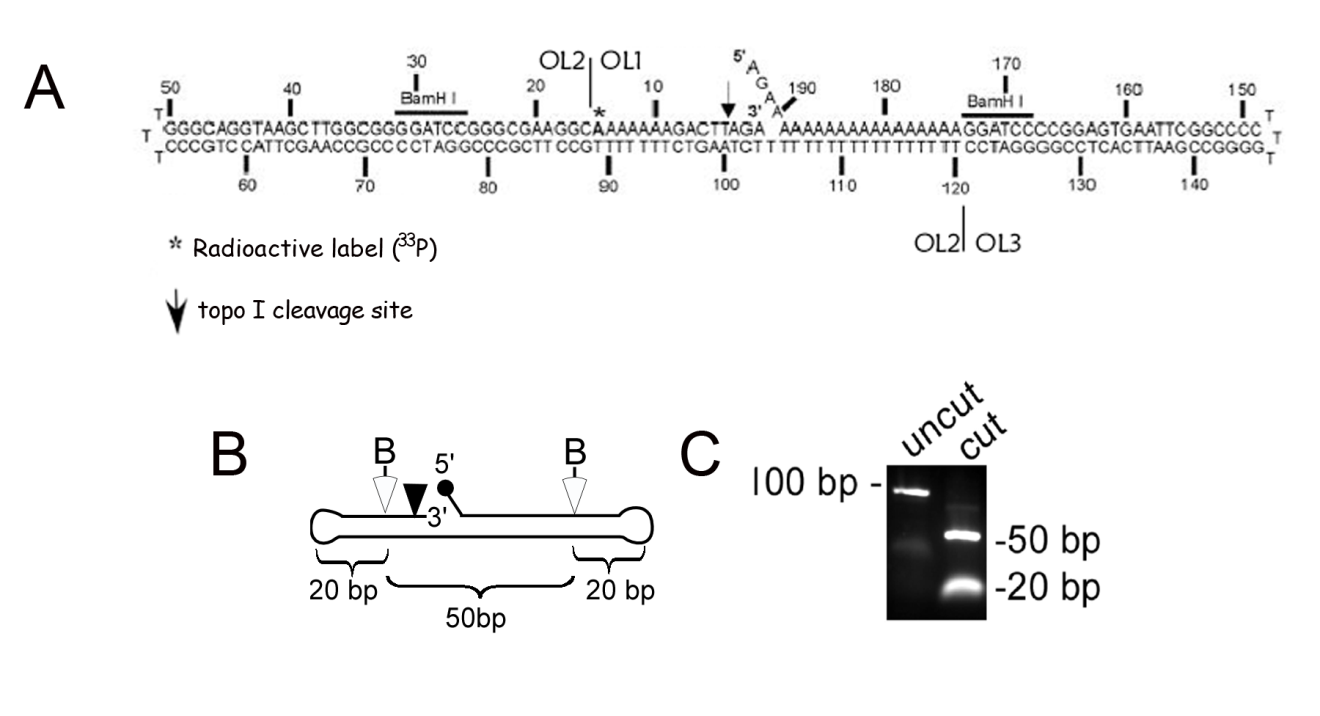


**Figure S2**

Supplement: Figure S2 — Description of suicide substrate used in Figure 2E . (A) The sequence of the synthetic suicide substrate (Figure adapted from reference (a) with permission from Oxford University Press). The substrate was a design taken from reference (a) that traps topoisomerase in a covalent complex with DNA. The substrate is a 193-base synthetic DNA that contains a 16-nucleotide topo I binding and cleavage sequence identified in the rDNA spacers of Tetrahymena and Dictyostelium (reference b). Religation of DNA and release of topo I cannot occur due to the lack of a 5′-hydroxyl group on the DNA. The substrate is formed by ligating 3 oligonucleotides (denoted OL1, OL2, OL3), then slowly annealing the ligated products to form a hairpin structure. OL1 provides the 3′ end of the ligated product and OL3 provides the 5′ end. OL1 was 5′-phosphorylated with [γ-33P]-ATP and T4 kinase prior to annealing and ligation to allow tracking of covalent protein–DNA complexes (indicated by *) and OL2 was 5′-phosphorylated with unlabeled ATP and T4 kinase prior to ligation. After 5′-phosphorylation of OL1 and OL2, the 3 oligos were precipitated, resuspended at 50 pmol/µl in 10 mM Tris (pH 8) and 1 mM EDTA, and 1 µl of each oligo was annealed in 100 µl of 10 mM sodium phosphate (pH 7) and 150 mM sodium chloride. The mixture was heated to 95°C to achieve complete denaturation, then slowly cooled to 25°C (2°C decrease per min). T4 polynucleotide ligase was added (1200 units; New England BioLabs), the mixture was incubated for an additional 3–4 days at 4°C, and the product was then treated with T4 kinase and ATP, as described in reference (a). (B) Schematic showing the final product, a double-stranded hairpin structure of 94 bp in length and phosphorylated at the 5′ end. The topo I cleavage site (▾) lies 3 nucleotides upstream of the engineered nick in which the 5′-hydroxyl group required for resealing is replaced by a phosphate group (•). (C) 10% TBE PAGE analysis validating the accuracy of annealing: w [file pone.0050427.s002.docx]
